# Comprehensive Analyses of the Effects of the Small-Molecule Inhibitor of the UHM Domain in the Splicing Factor *U2AF1* in Leukemia Cells

**DOI:** 10.21203/rs.3.rs-4477663/v1

**Published:** 2024-06-03

**Authors:** Xinrui Yuan, Mona Kazemi Sabzvar, Amol D. Patil, Krishnapriya Chinnaswamy, Kathryn L. Howie, Ramaraju Andhavaram, Borwyn Wang, Maxime A. Siegler, Arda Dumaz, Jeanne A. Stuckey, Seth J. Corey, Jaroslaw P. Maciejewski, Valeria Visconte, Chao-Yie Yang

**Affiliations:** 1 Department of Pharmaceutical Sciences, College of Pharmacy, University of Tennessee Health Science Center, Memphis, TN 38163, USA; 2 Life Sciences Institute, University of Michigan, Ann Arbor, MI 48109, USA; 3 Departments of Pediatrics and Cancer Biology, Cleveland Clinic, Cleveland, OH, 44195, USA; 4 Department of Chemistry, John Hopkins University, Baltimore, MD, 21218, USA; 5 Department of Translational Hematology and Oncology Research, Taussig Cancer Institute, Cleveland Clinic, Cleveland, OH, 44195, USA

**Keywords:** U2 small nuclear RNA auxiliary factor 1, U2AF2, UHM, ULM, RNA, HTRF, RNA splicing, endocytosis, clathrin, collagen, myeloid malignancies

## Abstract

Mutations in RNA splicing factor genes including *SF3B1, U2AF1*, *SRSF2*, and *ZRSR2* have been reported to contribute to development of myeloid neoplasms including myelodysplastic syndrome (MDS) and secondary acute myeloid leukemia (sAML). Chemical tools targeting cells carrying these mutant genes remain limited and underdeveloped. Among the four proteins, mutant U2AF1 (U2AF1^mut^) acquires an altered 3’ splice site selection preference and co-operates with the wild-type U2AF1 (U2AF1^wt^) to change various gene isoform patterns to support MDS cells survival and proliferation. *U2AF1* mutations in MDS cells are always heterozygous and the cell viability is reduced when exposed to additional insult affecting U2AF1^wt^ function. To investigate if the pharmacological inhibition of U2AF1^wt^ function can provoke drug-induced vulnerability of cells harboring *U2AF1*^*mut*^, we conducted a fragment-based library screening campaign to discover compounds targeting the U2AF homology domain (UHM) in U2AF1 that is required for the formation of the U2AF1/U2AF2 complex to define the 3’ splice site. The most promising hit (**SF1–8**) selectively inhibited growth of leukemia cell lines overexpressing*U2AF1*^*mut*^ and human primary MDS cells carrying *U2AF1*^*mut*^. RNA-seq analysis of K562-U2AF1^mut^ following treatment with **SF1–8** further revealed alteration of isoform patterns for a set of proteins that impair or rescue pathways associated with endocytosis, intracellular vesicle transport, and secretion. Our data suggested that further optimization of **SF1–8** is warranted to obtain chemical probes that can be used to evaluate the therapeutic concept of inducing lethality to *U2AF1*^*mut*^ cells by inhibiting the U2AF1^wt^ protein.

## Introduction

The recent update of the hallmarks of cancer broadened the repertoire of cellular properties acquired by tumor cells along their transformation paths to cancer.^1^ An overlooked characteristic of tumor cells is their manipulation of RNA processing to achieve the capability of the hallmark properties. In hematologic malignancies, frequent mutations of a set of genes that participate in RNA splicing have been discovered. In myelodysplastic syndrome (MDS) patients, gene sequencing studies of patients’ samples^2–11^ have revealed that genes encoding RNA splicing factors, *SF3B1, U2AF1*, *SRSF2*, *ZRSR2,* and *LUC7L2,*^2,12–14^ had a significantly higher mutational frequency and were associated with disease phenotypes (*e.g*., *SF3B1*, *U2AF1*). These genes are either a component of the spliceosome, such as *SF3B1*, or auxiliary splicing factors, including *U2AF1, SRSF2, ZRSR2*, that assist the execution of the splicing process where mutations in these genes was shown to have prognostic impact in myeloid malignancies.^15^ In the 5^th^ edition of the WHO Classification of Hematolymphoid tumors,^16,17^ *SF3B1* mutations have now replaced ring sideroblasts to become a subgroup in clinical diagnosis. Although *SF3B1* mutations are associated with favorable outcome in MDS^6,18^, *U2AF1* mutations^19^ give significant adverse risk and poor prognosis.^6,15^ *SRSF2* mutations are less frequent but cooccur with additional gene mutations to cause shorter overall survival in MDS.^15^ Despite the different prognosis in patient outcomes, targeting these mutant splicing factors offers a new therapeutic strategy to treat patients with hematological malignancies.^20,21^

**Figure 1.**
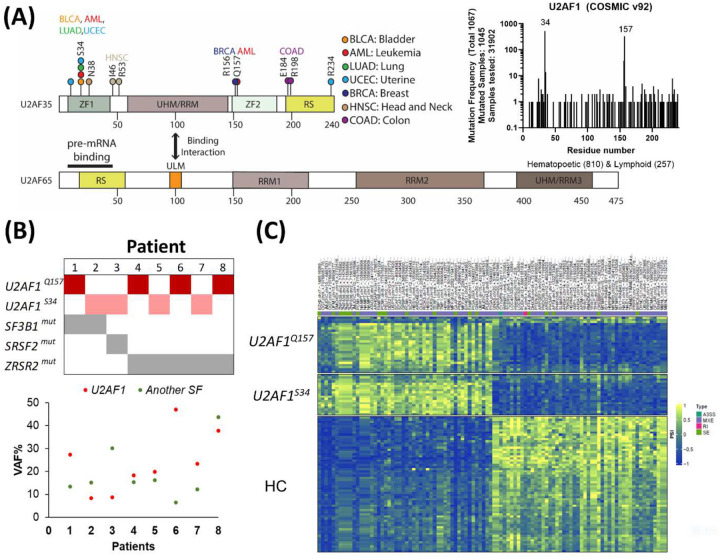
Mutational landscape and splicing profiles of U2AF1 hotspot mutations. (A) Mutational frequency in 33 types of tumors in samples collected from TCGA^39^ and COSMIC database. (B, upper panel) Co-occurring mutations of *U2AF1* with other splicing factor genes identified from the Cleveland Clinic cohort. A total of 202 *U2AF1* mutant patients was investigated with eight patients carrying mutations in another splicing factor. (B, lower panel) Variant allele frequency of the splicing factor mutations found in each patient (red color, *U2AF1* mutation; green color, mutation in another splicing factor (SF). (C) Splicing profile measured by percent spliced in (PSI) of patients with *U2AF1* mutation (S34 vs. Q157). A subset of genes was compared with healthy controls (HC), Different alternative splicing events were annotated (A3SS, alternative 3’ splice site; MXE, mutual exclusive events; RI, intron retention; SE, skipped exon).

In the analyses of MDS samples, patients carried either heterozygous missense mutations or hemizygous deletions with mutual exclusion in these genes.^8,15,20–22^ This implies that MDS cells may not tolerate acquisition of multiple mutant proteins involved in RNA splicing^5^ and rely on a wild-type copy of the mutant splicing factor for survival. The synthetic lethality^23,24^ to induce toxicity to tumor cells carrying splicing factor mutations may be applicable by targeting the remaining wild-type copy of the splicing factor or a second splicing factor without mutations. Indeed, studies have shown that leukemia cells carrying *SRSF2*^25,26^ and *U2AF1*^27^ mutations were sensitive to compounds impairing SF3B1 function. Genetic analysis of the disease progression of MDS patients further revealed that *U2AF1* mutations occur in the early clonal evolution^5^ and MDS patients carrying mutant *U2AF1* (*U2AF1*^*mut*^) are at increased risk to progress to secondary acute myeloid leukemia (sAML).^3,28,29^ Defective hematopoiesis associated with *U2AF1*^*mut*^ cannot be rescued by overexpression of wild-type *U2AF1* (*U2AF1*^*wt*^) in mice^30,31^ suggesting that *U2AF1*^*mut*^ might gain a new function or be neomorphic. Functionally, U2AF1 heterodimerizes with U2AF2 to associate with SF1 to form a SF1/U2AF2/U2AF1 complex that binds to the branch point sequence (BPS, with SF1), polypyrimidine track (with U2AF2), and the 3’ splice site (with U2AF1) on introns in the early step of pre-mRNA processing.^5,32^ Hotspot mutations (S34 and Q157) in the two zinc finger domains of U2AF1 (Fig. 1) directly impact the binding between U2AF1 and mRNA and have been shown to alter 3’ splice site recognition preferences^2,3,33,34^, resulting in mRNA transcript pattern changes. Downstream effects associated with U2AF1 mutations have been demonstrated in several *in vitro* mechanistic studies in cells. For example, *U2AF1*^*S34F*^ in mouse pro-B cell line (Ba/F3) generated a long *ATG7* transcript producing reduced levels of ATG7 and impaired autophagy to promote oncogenic transformation of MDS and AML.^35^ AML samples carrying U2AF1 mutations (S34F, R156H, and Q157P/R) were found to produce the pro-inflammatory long IRAK4 isoforms instead of the short IRAK4 that would maximize the oncogenic NF-κB pro-survival signaling.^36^ Induced pluripotent stem cells (iPSC) transfected with *U2AF1*^*S34F*^ were found to generate a long isoform of *GNAS* that activated ERK/MARK signaling potentially contributing to the neoplastic transformation of MDS.^37^ Growing evidence indicating that specific *U2AF1*^*mut*^-associated protein isoforms promote leukemia cell transformation strongly supports the notion that agents targeting U2AF1 will provide an important chemical tool to interrogate *U2AF1*^*mut*^ function and facilitate study of U2AF1 as a potential therapeutic target in *U2AF1* mutant myeloid neoplasia.

Previously, Chatrikhi *et al*. performed a compound library screen and identified a small molecule, NSC194308, that stabilized the SF1/U2AF2/U2AF1 complex and stalled pre-mRNA splicing at the early spliceosome assembly.^38^ Moreover, NSC194309 induced toxicity to K562 wild-type (K562-*U2AF1*^*wt*^) and K562 expressing *U2AF*^*S34F*^ (K562-*U2AF*^*S34F*^) at a 2–3-fold lower IC_50_ value. Isoforms changes of select genes in the K562-*U2AF1*^*wt*^ and K562-*U2AF1*^*S34F*^ were also determined. Here, we report the results of our library screening campaign that identified **SF1–8** inhibiting U2AF Homology Motifs (UHM) domain of U2AF1 (U2AF1-UHM) required for the binding between U2AF1 and U2AF2 and evaluated the effects of **SF1–8** on the viability and molecular pathways in *U2AF1*^*S34*^ mutant leukemia cells.

## Results

### Mutational landscape of *U2AF1* in tumor samples

Analysis of the whole-exome sequences in 33 tumor types in the Cancer Genome Atlas (TCGA) showed somatic mutations involving at least ten amino acids in U2AF1 across six tumor types besides leukemia (Fig.1A).^39^ More frequent mutations were found in U2AF1 S34. Analysis of the Catalogue of Somatic Mutations in Cancer (COSMIC) data showed that missense mutations of *U2AF1* accounted for 80% of tumor samples. S34 and Q157 were two hotspot mutations in hematopoietic and lymphoid tumors (Fig.1A). Our collection of studies in the Cleveland Clinic cohort showed *U2AF1* mutations at a frequency of 5–20% in MDS and sAML. Two hotspot mutations (S34; Q157) in *U2AF1* were predominant (Fig.1B). *U2AF1* mutations rarely co-occur with other common splicing factor mutations (*SRSF2*, *SF3B1*, *ZSZR2*).^40,41^ In a cohort of 202 MDS/AML patients at Cleveland Clinic, only eight patients acquired comutations of *U2AF1* and other splicing factors with variable variant allele frequency (VAF) in both hits (Fig.1B). Using curated RNA-sequencing data from samples with myeloid neoplasia (PMID: 36926651), we compared RNA splicing profiles of clonal cells with S34 (n=19) and Q157 (n=26) (VAF >10%, for both). The splicing profiles of *U2AF1*^S34^ and *U2AF1*^Q157^ overlapped for a subset of genes distinctive from healthy controls suggesting convergence rather than divergence of splicing outcomes (Fig.1C).

### Identification of SF1–8 from fragment library screening against U2AF1-UHM domain

**Figure 2.**
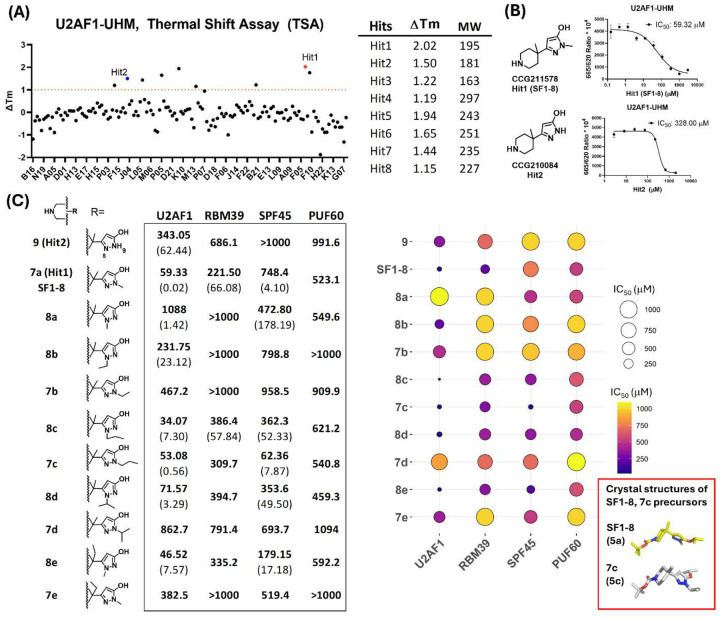
Fragment-based library screening against U2AF1-UHM using the TSA to discover hits, IC_50_ of SF1–8 and analogs for the U2AF1-, RBM39-, SPF45-, and PUF60-UHM. (A) The melting temperature changes (ΔTm) of U2AF1-UHM caused by the binding with compounds based on the thermal shift assay. The ΔTm of the top 8 hits and their molecular weight (MW). (B) Chemical structures of Hit1 and Hit2 and the IC_50_ values against U2AF1-UHM based on the representative titration cures from the HTRF assay. (C) The IC_50_ values are determined by the HTRF assay. The numbers in parentheses are standard deviation of 2 independent experiments for compounds with IC_50_ <300 μM. Each experiment is performed in duplicate. The crystal structures of **SF1–8**, **7c** precursors (**5a**, **5c** in **Scheme S1**) are shown in the insert box.

A previous small compound library screening study against the zinc finger (Zf) domains of U2AF1 did not identify hits.^38^ To develop a different targeting strategy, we assessed potential tractable binding sites in U2AF1 using computational analysis and determined that the UHM domain of U2AF1 was for identifying hits (**Fig.S1**).^42^ To discover hits, we screened the CCG fragment library from University of Michigan against the U2AF1-UHM domain using the Thermal Shift Assay (TSA). An initial single concentration screening at 200 μM led to identify 149 compounds that gave positive shifts of the melting temperature (ΔTm) in U2AF1-UHM. These compounds were subject to a secondary confirmatory screening in triplicate at 200 μM. Nine compounds showed ΔTm>1 °C in U2AF1-UHM (Fig.2A) in which CCG211578 and CCG210084 had a similar core structure with a difference of a methyl group (Fig.2B). CCG211578 gave the highest ΔTm at 2.02 °C whereas 1.50 °C of ΔTm was observed with CCG210084. Our screening data suggested CCG211578 and CCG210084 bound to stabilize U2AF1-UHM.

TSA data provided no information of the binding site in U2AF1-UHM. To identify the binding sites of both hits to U2AF1-UHM, we synthesized CCG211578 (named Hit1/**SF1–8**) and CCG210084 (named Hit2/**9** in Fig.2C) and asked if they inhibited the binding between U2AF1-UHM and U2AF2-ULM using our previously developed HTRF assay.^42^ We found both **SF1–8** and Hit2 inhibited the binding between U2AF1-UHM and U2AF2-ULM at the IC_50_ values of 59.33 ± 0.02 and 343.05 ± 62.44 μM respectively. The IC_50_ values of **SF1–8** and Hit2 correlated with the ΔTm values observed in TSA. We have reported that the conserved Trp binding site in U2AF1-UHM dominated the binding affinity between U2AF2-ULM and U2AF1-UH.^42^ Thus, data from TSA and HTRF assays suggested that both **SF1–8** and Hit2 may target the Trp binding site in U2AF1-UHM.

To determine the selectivity of **SF1–8** against other homologous UHM domains, we measured the IC_50_ values of **SF1–8** to RBM39-UHM, SPF45-UHM and PUF60-UHM using the HTRF assay.^42^ We found **SF1–8** had a 4–12 fold selectivity against three other UHM domains (Fig.2C), whereas Hit2 was a much weaker and less selective inhibitor. In the chemical synthesis (**Scheme S1**), methylation on the pyrazole group in **SF1–8** can occur at the N8 or N9 position (Fig.2C). Although the NMR spectra can be used to infer the methyl group position on the pyrazole, we determined the crystal structure of a **SF1–8** precursor to clearly confirm the methyl group is at N9 in **SF1–8** (Fig.2C). To further probe the interaction of **SF1–8** with U2AF1-UHM, we synthesized **SF1–8** analogs and developed the structure activity relationship (SAR) (Fig.2C). The SAR indicated that a methyl group at the N8 position (**8a**) decreased the activity against U2AF1-UHM. Although replacement of methyl with ethyl did not improve the activity to U2AF1-UHM, a propyl group at either the N8 (**8c**) or N9 (**7c**) position retained or had improved activity against U2AF1-UHM. When an isopropyl group was used, **8d** had a modest decreased activity against U2AF1. A longer alkyl group was tolerated at the N8 position but not at the N9 position. We also replaced the methyl group on the piperidine ring with an ethyl group and found **8e** had a comparable IC_50_ value with **SF1–8**. Compounds (**7c**, **8c**, **8d**, **8e**) that had comparable IC_50_ values to **SF1–8** were generally more selective against three other UHM domains except **7c** that had a dual inhibitory activity against U2AF1 and SPF45 (Fig. 2C). The crystal structure of a **7c** precursor experimentally determined from SCXRD again confirmed the location of the propyl group (Fig. 2C). The biophysical binding data indicated that **SF1–8** and **8c** preferentially inhibited U2AF1-UHM against other UHM domain proteins.

### Activity of U2AF1-UHM inhibitors in model cell lines and primary cells from MDS patients

**Figure 3.**
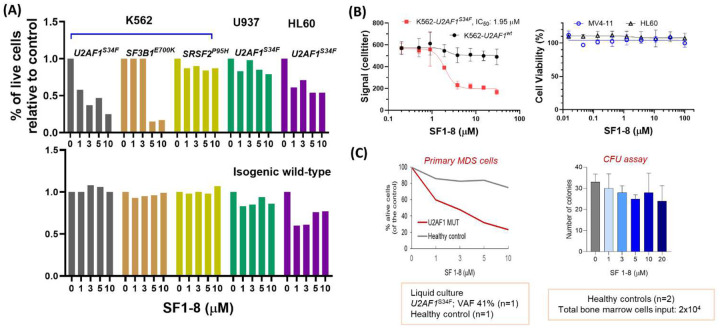
Growth inhibition activities of SF1–8 in U2AF1, SF3B1, SRSF2 mutant, wild-type leukemia cell lines (A) Dose dependent growth inhibition of *U2AF1*^*S34F*^*, SF3B1*^*E700K*^*, SRSF2*^*P95H*^ mutant and isogenic wild-type cell lines by the treatment of **SF1–8**. (B) Titration of **SF1–8** against K562-*U2AF1*^*wt*^, K562-*U2AF1*^*S34F*^, MV4–11, HL60 cells using the CellTiter-Glo assay. (C) Toxicity of **SF1–8** in human primary MDS cells (*U2AF1*^*S34F*^) and healthy bone marrow cells.

Because the biophysical assay suggested that **SF1–8** was more effective and selective against U2AF1-UHM, we proceeded to evaluate **SF1–8** in cell lines. Previous studies that introduced *U2AF1*^*S34F*^ in lung epithelial (HBEC3kt)^43^, leukemia (K562)^38,44,45^, and iPSC^37^ cell lines have validated the function of *U2AF1*^*S34F*^ in these engineered cell lines while mutation of Q157 is less studied. We therefore focused our study on *U2AF1*^*S34F*^ and examined the effects of **SF1–8** on a panel of leukemia cell lines including K562, U937, HL60 with isogenic overexpression of *U2AF1*^*wt*^ or *U2AF1*^*S34F*^, K562 with isogenic overexpression of *SRSF2*^*wt*^ or *SRSF2*^*P95H*^, and K562 with Crispr/Cas9 edited *SF3B1*^*wt*^ or *SF3B1*^*E700K*^ (denoted as K562-*U2AF1*^*wt*^, K562-*U2AF1*^*S34F*^, U937-*U2AF1*^*wt*^, U937-*U2AF1*^*S34F*^, HL60-*U2AF1*^*wt*^, HL60-*U2AF1*^*S34F*^, K562-*SRSF2*^*wt*^, K562-*SRSF2*^*P95H*^, K562-*SF3B1*^*wt*^, K562-*SF3B1*^*E700K*^). We found that **SF1–8** selectively decreased the viability of K562-*U2AF1*^*S34F*^, K562-*SF3B1*^*E700K*^ but not their isogenic wild-type cells (Fig.3A). Although **SF1–8** had no activity in K562-*SRSF2*^*wt*^, K562-*SRSF2*^*P95H*^, U937-*U2AF1*^*wt*^, and U937-*U2AF1*^*S34F*^, **SF1–8** reduced cell viability in both HL60-*U2AF1*^*wt*^ and HL60-*U2AF1*^*S34F*^ similarly. The dosetitration experiment gave an IC_50_ value of 1.98 μM in K562-*U2AF1*^*S34F*^ but showed no activity in K562-*U2AF1*^*wt*^ (Fig. 3B). In the native leukemia cell lines (MV-4–11 and HL-60) carrying *U2AF1*^*wt*^, **SF1–8** showed no activities up to 100 μM (Fig.3B). The data obtained from the engineered leukemia cell lines prompted us to query the activity of **SF1–8** in primary MDS cells. We plated bone marrow cells from an MDS specimen that harbored *U2AF1*^*S34F*^ (VAF, 41%) and found **SF1–8** dose-dependently reduced the viable cells to 20% at 10 μM (Fig.3C). In contrast, as much as 10 μM **SF1–8** did not significantly decrease 20% of viable cells in healthy control cells. **SF1–8** showed no significant toxicity to the bone marrow cells of healthy control from 1 to 20 μM in the colony formation assay (Fig.3C).

### Transcriptomic changes in K562-*U2AF1*^*S34F*^ cells treated with SF1–8

**Figure 4.**
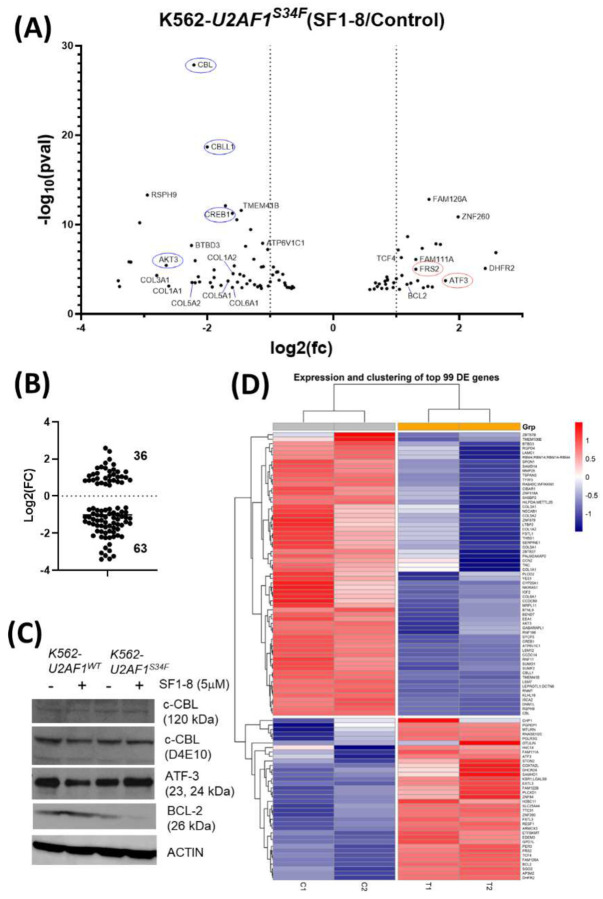
RNA-seq experiment of K562-*U2AF1*^*S34F*^ cells treated with SF1–8 and the western blot of select genes. (A) Volcano plot of genes significantly affected by **SF1–8**. (B) Number of genes significantly up- and down-regulated by **SF1–8**. (C) Western blots of c-CBL, ATF3, and BCL-2 in K562-*U2AF1*^*wt*^, K562-*U2AF1*^*S34F*^ treated with **SF1–8**. (D) Heatmap of top 99 differentially regulated genes in K562-*U2AF1*^*S34F*^ treated with **SF1–8.**

To understand the underlying mechanisms of the differential effects of **SF1–8** on K562-*U2AF1*^*S34F*^, we performed an RNA-seq experiment on K562-*U2AF1*^*S34F*^ cells treated with **SF1–8** and control (Fig.4). In the **SF1–8** treated K562-*U2AF1*^*S34F*^ cells, we found 36 up- and 63 down-regulated genes in the top 99 differentially expressed genes (Figs.4B, 4D). Down-regulated genes included *c-CBL* (E3 ligase), *CBLL1* (E3 ligase), *CREB1* (transcription factor), *AKT3* (kinase regulating signaling mediated by growth factor) and a set of collagens, including *COL1A1*, *COL1A2*, *COL3A1*, *COL5A1*, *COL5A1*, and *COL6A1* (Fig.4A). Up-regulated genes included *ATF-3* (stress response transcription factor), *FRS2* (growth factor binding protein), *DHFR2* (folate metabolism in mitochondria), and *BCL-2* (anti-apoptotic). When the 99 genes were mapped to KEGG pathways^46^, the top six enriched pathways were protein digestion and absorption, extracellular matrix-receptor interaction, focal adhesion, PI3K-Akt signaling pathway, human papillomavirus infection, and AGE-RAGE signaling pathway (**Fig.S2**). To determine if the protein levels of these genes were affected by their mRNA changes, we performed the western blots of select genes. An increase of ATF-3 was confirmed, but we found no change of c-CBL and decreased BCL2 (Fig.4C). Because of the role U2AF1 in RNA splicing, we asked if analysis of the isoform pattern changes may provide insight to explain the different observations between RNA-seq and western blot data. In the transcript analysis, we identified low FPKM numbers of three *BCL2* transcripts (**Fig.S3**) that explained the relatively low BCL2 protein levels in K562. A significant increase of the *BCL-2*b transcript was also found. *BCL-2*b possessed an apoptotic function and was reported to have reduced mRNA and proteins stability than the canonical isoform^47^ that partly explained the decreased BCL-2 protein level in western blot despite mRNA increased. Besides BCL2, BCLXL (encoded by *BCL2L1*) and MCL1 have isoforms (BCL-XS and MCL-1S) that had apoptotic functions. K562 cells expressed only the anti-apoptotic *MCL1* transcript and the expression was not affected by **SF1–8** (not shown). Similarly, **SF1–8** did not significantly change the isoform pattern of *BCL2L1* gene (**Fig.S3**). Another frequently studied gene involved in apoptosis regulation is survivin.^48,49^ Multiple transcripts of survivin have been discovered and possess either anti-apoptotic or apoptotic functions.^50^ In the K562-*U2AF1*^*S34F*^ cells, we found **SF1–8** markedly increased the transcript (*Survivin-2a*) that had the apoptotic function (**Fig.S3**).

### Isoform pattern changes in K562-*U2AF1*^*S34F*^ cells treated with SF1–8

**Figure 5.**
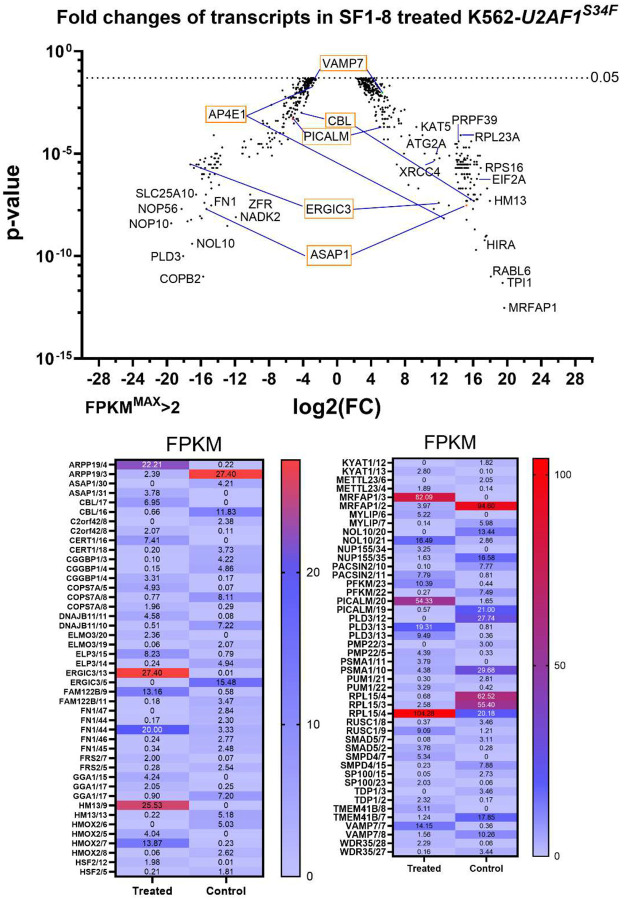
Genes exhibit changes in expression patterns in transcripts containing different number of exons from the RNA-seq experiment. Average FPKM values are displayed. Genes involved with endocytosis and vesicle transport discussed in the text are highlighted with boxes in the volcano plot. In the heatmap, each gene is annotated by the gene name followed by the number of exons in each transcript.

To identify additional players, we examined transcripts that were significantly changed by **SF1–8** in K562-*U2AF1*^*S34F*^ cells. A total of 1034 transcripts significantly changed their mRNA expression levels after **SF1–8** treatment in the K562-*U2AF1*^*S34F*^ cells. To filter potential false-positive hits, we included transcripts that had at least one sample containing FPKM >2. In this set of transcripts, we first identified for genes that changed expression patterns in ≥2 transcripts carrying different numbers of exons and identified 39 genes (Fig.5). Additionally, eight genes (*BCOR, CENPE, CENPK, ZBTB7B, HNRNPC, HNRNPH1, MRKN,* and *PARN*) showed a same pattern of changes in two transcripts (either both up- or down-regulated) carrying different number of exons, and 32 genes exhibited variable changes of expression patterns in multiple transcripts containing the same number of exons **(Fig.S4)**.

Changes of isoform pattern in genes may alter their functions in cells; therefore, we focused on studying the 39 genes that changed isoform pattern. To understand if these genes interact with each other, we used the STRING database to map the interactome of the 39 genes (**Fig.S5**). A few network nodes of interaction were detected. They included the network interaction of a) *FRS2, CBL, FN1, ASAP1, PACSIN2*; b) *VAMP7, PICALM, PLD3* and paired interaction nodes in *NUP155/SP100, DNAJB11/HSF2, PSMA1/RPL15, NOL10/ELP3* and *ELMO3/WDR35*.

### Isoform pattern changes of multiple genes by SF1–8 affected endocytosis and vesicle transports in K562-*U2AF1*^*S34F*^ *cells*.

**Figure 6.**
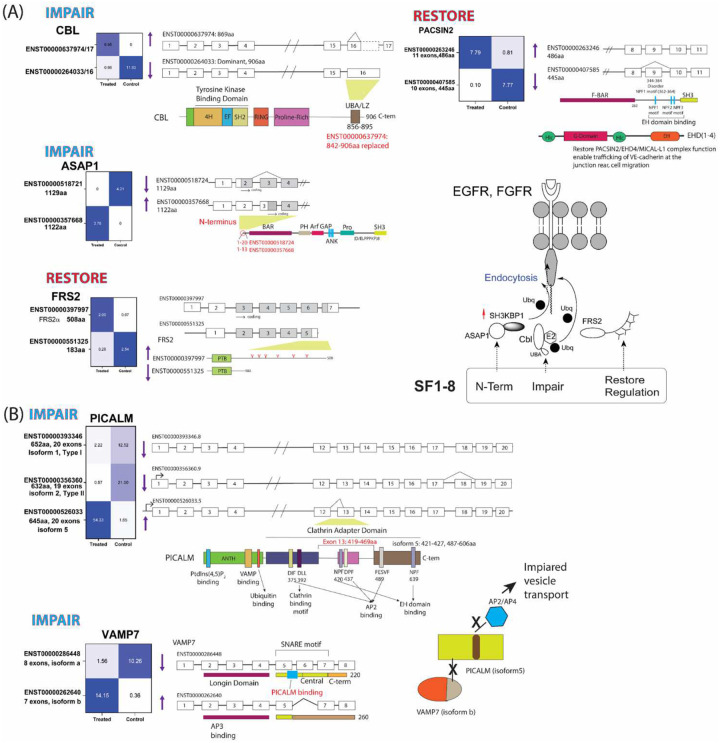
Impacts on the (A) CBL-FRS2-ASAP1-PACSIN2 and (B) PICALM-VAMP7 network interaction by the treatment of SF1–8 in K562-*U2AF1*^*S34F*^ *cells*. The exon selection and the effect on the protein domains in each gene are depicted. The up and down arrows indicate the up- or down-regulation caused by the **SF1–8** treatment. Summary of the potential impact on the signaling associated with the isoform changes is illustrated in both cases.

We first analyzed the isoform pattern changes of the genes in the largest network node that included *FRS2, CBL, FN1, ASAP1,* and *PACSIN2* (Fig.6A). **SF1–8** treatment led to an expression change of *CBL* from the dominant transcript (encoding 906 aa) to an isoform, ENST00000637974, (encoding 869aa) that contained different amino acids in the C-terminal ubiquitin-associated (UBA) domain of *CBL*.^51^ The alternative UBA domain in ENST00000637974 may adopt a disordered conformation according to AlphaFold^52^ and impaired the homodimerization of CBL^51^ that is required for its function in cells.^51,53,54^ Both isoforms of CBL may be indistinguishable in our western blot experiment because the C-terminal domain of c-CBL is not the epitope of our antibody (Fig.6C). For *ASAP1*, a dominant isoform (1129 aa) was switched to an isoform lacking part of the N-terminus preceding the BAR domain.^55^ For *FRS2*, we found that the endogenous short isoform (encoding 183aa) was replaced by a dominant isoform (encoding 383 aa) that contained multiple regulatory tyrosine phosphorylation sites in the C-terminus.^56^ A similar switch of *PACSIN2* from an isoform that contained exon 9 skipped truncated EH domain binding region to the dominant isoform was found. Functionally, ASAP1 and FRS2 have been reported to facilitate the recruitment of ubiquitinated c-CBL to the membrane EFGR and FGFR and promote the ubiquitination and endocytosis of EGFR and FGFR.^57–59^ Changes in the isoform patterns induced by **SF1–8** may increase the regulation of FRS2, perturb the ASAP1 BAR domain mediated binding, and disable the UBA domain in c-CBL, collectively limiting or impairing the endocytosis of the growth factors such as EFGR and FGFR. On the other hand, exon 9 in *PACSIN2* is mapped to a NPF(Asn-Pro-Phe)1 motif that interacts with the EH domain containing proteins.^60^ PACSIN2 also contains an F-BAR domain^61^, similar to the function of the BAR domain in ASAP1. The BAR domains are involved with creating membrane curvature and membrane dynamics.^60,62,63^ Restoration of PACSIN2 to the dominant isoform by **SF1–8** however may improve its function to participate in membrane dynamics and receptor-mediated endocytosis.^64^ The changes in PACSIN2 may serve as a rescuing feedback loop.

The second network node involved *PICALM*, *VAMP7*, and *PLD3* (Fig.6B). PICALM has been reported to be an important gene contributing to leukemogenesis and expression pattern change was observed in MDS/AML patients harboring *U2AF1* mutation.^65^ Our analysis of the transcripts of *PICALM* and *VAMP7* showed that isoforms switch in *PICALM* and *VAMP7* led to deletion of NPF and DPF motifs in *PICALM* that interact with AP2 and a truncated PICALM binding domain in *VAMP7*.^66^ Changes in the *PICALM* and *VAMP7* isoforms may result in decreased interaction between PICALM, VAMP7 and AP2 that is important for clathrin-coated vesicle formation and clathrin-mediated endocytosis.^67,68^ Another function of VAMP7 is to participate in trafficking late endosome to endolysosome and interact with SNARE to direct vesicles from Golgi to late endosome.^69–72^ Impairment of VAMP7 function may affect the vesicles sorting which involved PLD3. Although changes of three *PLD3* transcripts were found, they encoded the same protein and total *PLD3* mRNA was insignificantly downregulated by **SF1–8**.

**Figure 7.**
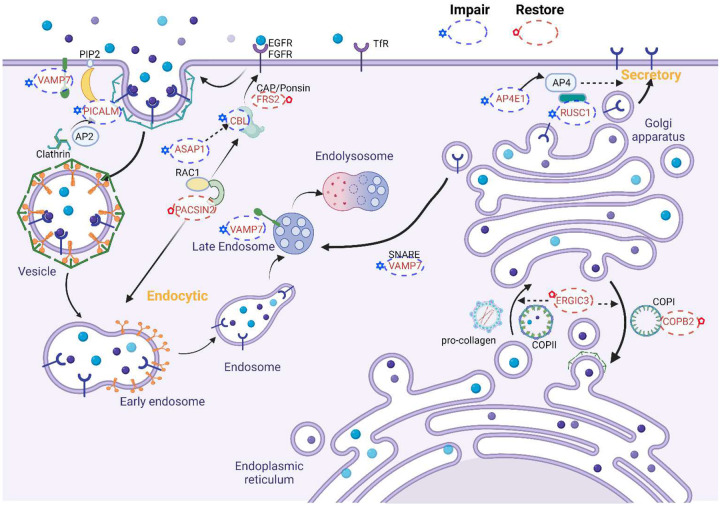
Summary of impacts on the clathrin-mediated endocytosis, vesicle transport between organelles, and the secretory pathway impacted by the treatment of SF1–8 in K562-*U2AF1*^*S34F*^ cells. The figure was prepared using BioRender.com.

We also identified additional genes that participated in vesicle transport and protein excretion. For example, ERGIC3 (Fig.5B) and COPB2 (**Fig.S4B**) regulated COPI and COPII vesicle transports between Golgi and endoplasmic reticulum (ER).^73–75^ For both genes, **SF1–8** switched the isoforms to their dominant isoforms that may improve the protein transport between organelles (**Fig.S6**). Further, RUSC1 interacts with AP4 to regulate vesicle secretion.^76^ Four transcripts of *RUSC1* **(Fig.S6)** were detected and the most significantly upregulated was the transcript (ENST00000368349) encoding an isoform lacking the N-terminal 468 aa with an unknown function. Two transcripts of the same number of exons in *AP4E1* also changed their expression pattern (**Fig.S6**). The dominant *AP4E1* transcript switched to an isoform with a truncated N-terminal clathrin/cooctamer adapter domain that may impair the trans-Golgi network vesicle transport^77^ and secretion function of AP4E1.^78^ The isoform changes in *RUSC1* and *AP4E1* by **SF1–8** treatment may impair normal vesicle secretion. A summary of the discussed genes involved with the endocytosis, vesicle transport, and protein secretion pathways was illustrated in Fig. 7.

### Impact on isoform pattern changes of genes involved in proteosome, and in MDS patients harboring splicing factors mutation were found in K562-*U2AF1*^*S34F*^ cells treated with SF1–8

Another interaction node was between *PSMA1* and *RPL15*. PSMA1 is a subunit of S20 proteosome and responsible for recruiting PSMC3 ATPase to facilitate the digestion of ubiquitinated proteins. **F1–8** treatment led to reduce the endogenous *PSMA1* isoform lacking the first 12 amino acid (ENST00000530457) to the dominant *PSMA1* isoform encoding 263aa (ENST00000396394) and increased a longer isoform encoding 269aa (ENST00000418988). The effect may improve the proteosome activity. Although expressions of three *RPL15* transcripts were detected, they all encoded the same *PFKM)* affected by *SF3B1*, *SRSF2*, and *U2AF1* mutations in MDS patients reported previously.^65^ In our K562-*U2AF1*^*S34F*^ model cell line, **SF1–8** markedly altered the isoform patterns of *PICALM, ERGIC3, RUSC1* discussed previously (Fig.5), *BCOR*, and *MRKN1* (**Fig.S4**). For *BCOR* and *MRKN1*, our RNA-seq analysis showed two isoforms of *BCOR* and *MRKN1* were downregulated and upregulated respectively in K562-*U2AF1*^*S34F*^ treated with **SF1–8**.

## Discussion

Next generation sequencing of patients’ samples has led to discover a set of splicing factors that are frequently mutated in the development of MDS and AML. The clinical observation that heterozygous *U2AF1* mutations are acquired in early clones suggests that mutant *U2AF1* may play an important role in the initiation phase of disease development by cooperating with the wild-type allele. Pharmacological intervention to the evolution of *U2AF1* mutant clone especially in MDS may offer an attractive therapeutic approach to mitigate the disease progression and improve patient treatment. Our strategy to abrogate the growth of the *U2AF1* mutant cancer cells is to target U2AF1-UHM that binds with U2AF2 to form a dimer recognizing the 3’ splice site in pre-mRNA splicing. Although inhibition of the U2AF1-UHM domain affects both the wild-type and mutant U2AF1, we hypothesized that the outcome may introduce a second alteration on the spliceosome assembly via U2AF1 to induce synthetic lethality to the U2AF1 mutant clones.

To investigate the hypothesis, we performed a library screening against U2AF1-UHM and discovered **SF1–8**. Using the HTRF binding assay, we verified that **SF1–8** inhibited the binding between U2AF1-UHM and U2AF2-ULM. We further characterized that **SF1–8** was selective against three other UHM-containing proteins (RBM39, SPF45, and PUF60). The SAR study of **SF1–8** analogs led to identify several compounds giving similar or slightly improved activity than **SF1–8**, but **SF1–8** remained more selective against RBM39, SPF45, and PUF60. Although the activity of **SF1–8** to U2AF2-UHM was not determined, a relatively selective U2AF2-UHM inhibitor, UHMCP1, was recently reported^79^ and characterized.^80^ Improvement of the activities of UHMCP1 and our U2AF1-UHM inhibitors will provide selective chemical probes to interrogate the role of each UHM containing protein in RNA splicing.

Our cellular activity study showed that **SF1–8** selectively reduced viability of K562-*U2AF1*^*S34F*^ but not K562-*U2AF1*^*wt*^ cells. Similar selective growth inhibition was observed between K562-*SF3B1*^*E700K*^ and K562-*SF3B1*^*wt*^ cells but not between K562-*SRSF2*^*P95H*^, U937-*U2AF1*^*S34F*^, and HL60-*U2AF1*^*S34F*^ and their isogenic wild-type cells. Of note, a compound, NSC194308 that stabilized SF1/U2AF2/U2AF1 complex, was previously shown to exhibit 2-fold selectivity between K562-*U2AF1*^*S34F*^ and K562-*U2AF1*^*wt*^.^38^ Although K562 cell line has been used as a model system in previous studies,^27,31,36,38,43,81–84^ the effects of U2AF1^S34F^ on the growth and survival in U937 and HL60 have not been characterized. To overcome the deficiency that no known MDS or AML cell lines acquired endogenous *U2AF1*^*S34F*^ mutation for inhibitor evaluation, we tested **SF1–8** in primary MDS patient cells carrying *U2AF1*^*S34F*^ (VAF, 41%) and found **SF1–8** also inhibited the proliferation of the primary cells.

In the RNA-seq analysis of K562-*U2AF1*^*S34F*^ treated with **SF1–8**, we observed down-regulation of CBL and a subset of collagens genes. Analysis of mRNA transcripts led to identify pattern changes of 39 transcripts in genes expressing different number of exons. The gene network interaction analysis revealed sets of genes participating in endocytosis, clathrin-mediated coat formation, intra-cellular vesicle transports between organelles and vesicle secretion were affected by **SF1–8**. The isoform pattern changes of these genes likely resulted in impairment of endocytosis (*EGFR*), clathrin-mediated coat formation (*PICALM*), endolysosome transport (*VAMP7*), and vesicle secretion (*RUSC1, AP4E1*) (Fig.7). Compensating effects on this pathway were observed in the restoration of the canonical transcripts in *PACSIN2* (endocytosis). Two genes, *ERGIC3* and *COPB2*, also switched to the dominant isoforms that may improve the COPI and COPII mediated vesicle exchange between ER and Golgi.^85^ COPB2 is a subunit of COPI^75^ that recycles proteins from Golgi to ER. Restoration of the canonical functions in ERGIC3 and COPB2 may be a feedback mechanism to manage impaired protein secretion associated with RUSC1 and AP4E1. Whether the altered activity in vesicle transport between ER and Golgi plays a role in upregulation of stress response genes, such as ATF3, remains to be studied.

Although we determined the impacts of **SF1–8** on the growth inhibition and transcriptome of K562-*U2AF1*^*S34F*^ cells in this work, there are limitations to our findings. Despite the preferentially inhibition of **SF1–8** to U2AF1-UHM, the activity and selectivity of **SF1–8** need to be improved. Activity of **SF1–8** against other potential targets in cells was not fully determined and may contribute to the activity observed in our study. Although RNA-seq data elucidated complex isoform transcript changes induced by **SF1–8**, follow-up experiments using PCR and western blot experiments (limited by suitable antibodies to detect specific protein isoforms) will be important to confirm the underlying mechanisms that reduced the viability of K562-*U2AF1*^*S34F*^ cells. Finally, murine models recapitulating heterozygous *U2AF1* mutations under development^31^ may be important tools to assess this therapeutic approach in future studies. Despite our demonstration of the feasibility to selectively target U2AF1-UHM with small-molecule inhibitors, the potential of advancing chemical tools targeting *U2AF1* mutant cells to drug development in pre-clinical evaluation remains to be fully investigated.
